# Sternocleidohyoid muscle: an unreported variant of cleidohyoid muscle

**DOI:** 10.1007/s00276-021-02682-0

**Published:** 2021-02-01

**Authors:** Satheesha B. Nayak, Surekha D. Shetty

**Affiliations:** Melaka Manipal Medical College (Manipal Campus), Manipal Academy of Higher Education, Madhav Nagar, Manipal, 576104 Karnataka India

**Keywords:** Infrahyoid muscles, Sternohyoid, Sternothyroid, Omohyoid, Cleidohyoid

## Abstract

Sternohyoid, sternothyroid, omohyoid, and thyrohyoid muscles are collectively known as infrahyoid muscles. These muscles frequently show variations in their attachments. Here, an extremely rare variant muscle belonging to this group has been presented. During cadaveric dissection for undergraduate medical students, an additional muscle was found between sternohyoid and superior belly of omohyoid muscles bilaterally in a male cadaver aged approximately 70 years. This muscle took its origin from posterior surface of the manubrium sterni, capsule of the sternoclavicular joint and the posterior surface of the medial part of the clavicle. It was inserted to the hyoid bone between the attachments of sternohyoid and superior belly of omohyoid muscles and was supplied by a branch of ansa cervicalis profunda. There is no report on such a muscle in the literature and it could be named as “sternocleidohyoid muscle”. Knowledge of this muscle could be useful in neck surgeries.

## Introduction

The infrahyoid muscles act on the thyroid cartilage of the larynx and the hyoid bone and their actions help in speech, swallowing and mastication. Infrahyoid muscles include sternohyoid and omohyoid superficially and sternothyroid and thyrohyoid as deeper structures. The omohyoid consists of superior and inferior bellies joined by an intermediate tendon, while sternohyoid, sternothyroid and thyrohyoid have only one muscle belly. Ansa cervicalis profunda innervates the muscles [17]. Infrahyoid muscles differ significantly in the extent of their development [2]. The infrahyoid muscles are hypaxial derivatives and are formed by myoblasts of cervical myotomes [13].

Variations in these muscles were identified during 19th century [19]. A few authors tried classifying the anatomical variation that contains muscle inserting from the clavicle into the hyoid bone. During 1912 Loth classified it as cleidohyoid muscle [24]. Such muscles were described as cleidohyoideus accessorius muscle in the presence of intact omohyoid by Sushma et al. [20]. Unilateral [5] and bilateral [18] “Cleidohyoideus accessorius” have also been reported.

Omohyoid muscle shows variations very frequently. One such variant of this muscle is a muscle presenting as an additional muscle belly extending between clavicle and hyoid bone [8] Other reported variants of the omohyoid muscle include absence of its superior belly [22], duplicated superior belly [11, 15] coursing deep to the internal jugular vein, short omohyoid [15] and existence as the variant cleidohyoideus muscle [3, 5].

Variations related to sternohyoid or sternothyroid muscles are seen in the literatures [6, 14, 16]. Variations in sternohyoid muscles are double or absent, augmented by a clavicular slip, or interrupted by a tendinous intersection [2]. Absence of sternal attachment is more common than the absence of clavicular attachment [17]. We found a unique muscle among the infrahyoid muscles during our dissection classes. We have thoroughly reviewed the literature about these muscles and have correlated its embryological and clinical importance in this case report.

## Case report

**Fig. 1:**
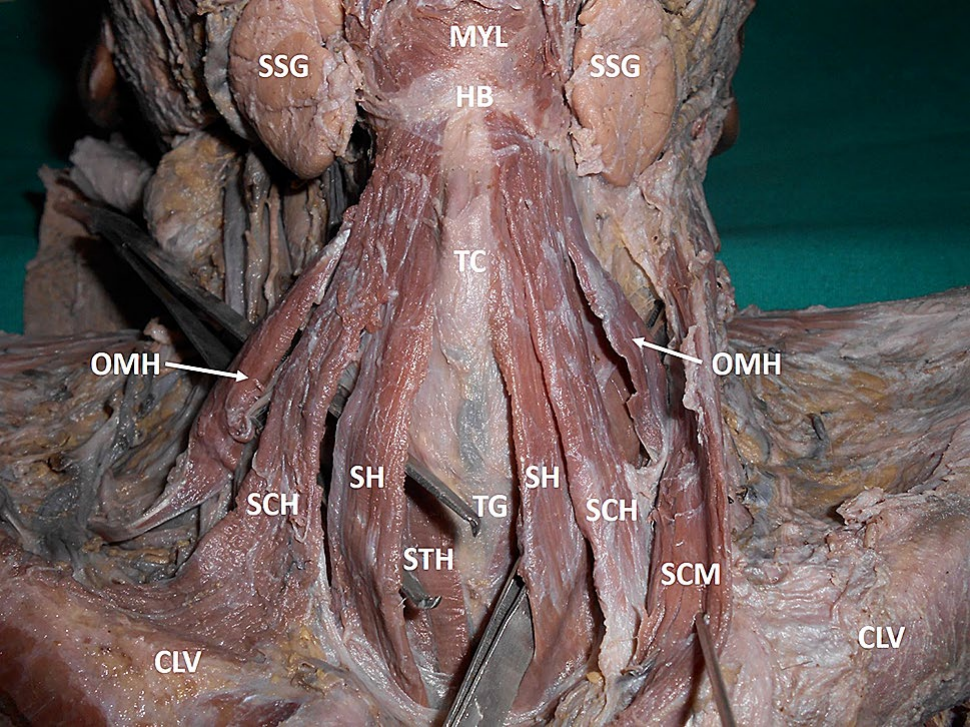
Dissection of the front of the neck showing the variant muscle. MYL: mylohyoid, SSG: submandibular salivary gland, HB: hyoid bone, TC: thyroid cartilage, OMH: superior belly of omohyoid, SCH: sternocleidohyoid muscle, SH: sternohyoid muscle, STH: sternothyroid, TG: thyroid isthmus, LV: clavicle, SCM: sternocleidomastoid muscle

**Fig. 2:**
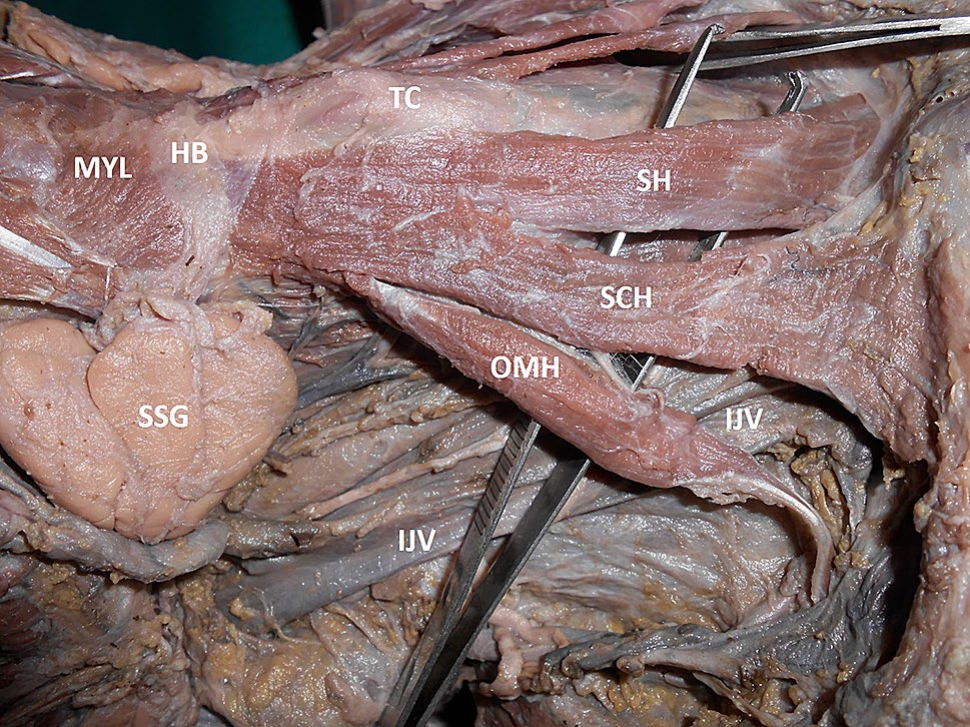
Right lateral view of the neck showing the variant muscle. MYL: mylohyoid, SSG: submandibular salivary gland, HB: hyoid bone, TC: thyroid cartilage, OMH: superior belly of omohyoid, SCH: sternocleidohyoid muscle, SH: sternohyoid muscle, IJV: internal jugular vein

**Fig. 3:**
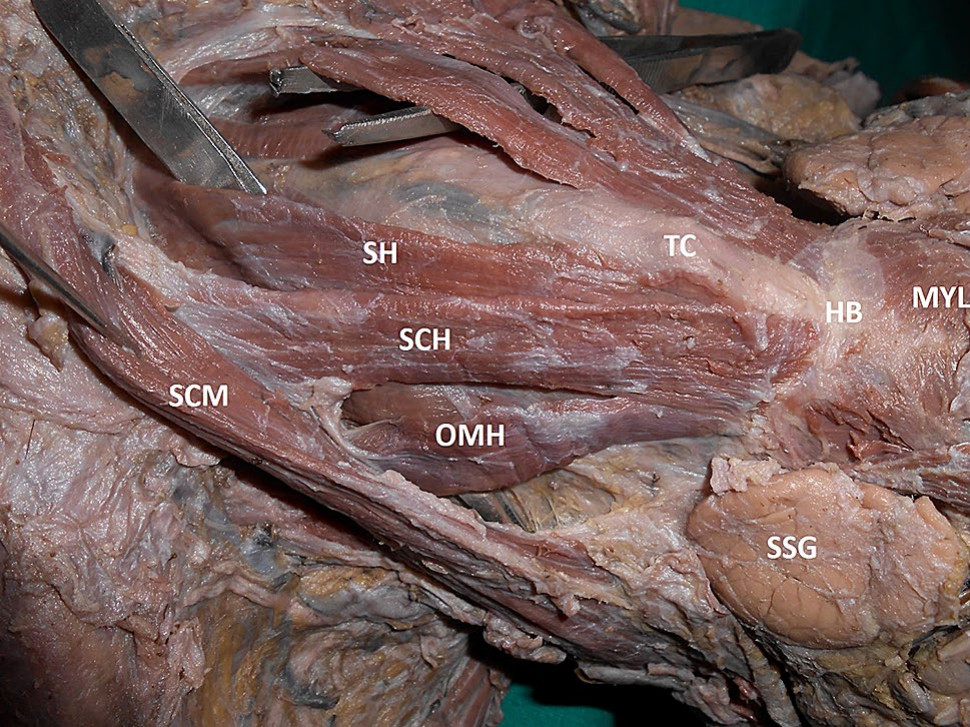
Left lateral view of the neck showing the variant muscle. MYL: mylohyoid, SSG: submandibular salivary gland, HB: hyoid bone, TC: thyroid cartilage, OMH: superior belly of omohyoid, SCH: sternocleidohyoid muscle, SH: sternohyoid muscle, STH: sternothyroid, SCM: sternocleidomastoid

During cadaveric dissection for undergraduate medical students, an additional muscle was found between sternohyoid and superior belly of omohyoid muscles bilaterally in a male cadaver aged 70 years, which was donated to the university for research and educational purpose. The additional muscle found, was slightly broader than sternohyoid muscle. It was placed in a superficial plane, with superior belly of omohyoid than the sternohyoid muscle. This muscle took its origin from posterior surface of the manubrium sterni, capsule of the sternoclavicular joint and the posterior surface of the medial part of the clavicle. It was inserted to the hyoid bone between the attachments of sternohyoid and superior belly of omohyoid muscles and was supplied by a branch of ansa cervicalis profunda. There were no other notable variations in the neck. The variant sternocleidohyoid muscle has been shown in Figs. 1, 2 and 3).

## Discussion

The surgical approaches like in thyroidectomy, tracheotomy, and removal of masses from the neck needs a good knowledge of normal anatomy and the variations of muscles in this region. Knowledge of variations of infrahyoid muscles is useful even in diagnostic procedures.

Some infrahyoid muscles originating from the clavicle and getting inserted into the hyoid bone have been describe in earlier studies [8, 10]. One such muscle is the cleidohyoideus accessorius muscle [4, 7, 18, 21]. Omohyoid muscle is the most variable muscle among the infrahyoid muscles. Either one or both its bellies may be absent [24]. Apart from the three known infrahyoid muscles, a fourth muscle originating from clavicle and getting inserted to the hyoid bone has been reported [25].

Variants of the omohyoid muscle are surgically and functionally important because of its close relationship with the great vessels and brachial plexus. Muscle contraction has a direct influence on internal jugular vein because of direct attachment of the tendon to its wall. Sternothyroid muscle is also known to show variations. Its absence, doubling, presence of a membranous tendon, and development of a cruciate pattern between the muscles of two sides are its reported variations [1, 14, 24]. Sternohyoid muscle flap for laryngeal reconstruction improves the quality of life in supracricoid laryngectomy without reducing the oncologic effectiveness of surgery [27]. Presently the use of infrahyoid myocutaneous flaps is a well-established alternative for reconstructing surgical defects. Supernumerary muscles, as double omohyoids, can be utilized for muscle reconstruction. Infrahyoid myocutaneous flaps have been used for the reconstruction of the tongue after resection of lingual carcinoma [26]. During central venous catheterization approach for internal jugular vein cannulation, appropriate anatomical knowledge is essential for anesthetists. Occurrence of an accessory muscle in the infrahyoid region have its implications during diagnostic and surgical procedures. Presence of additional muscle in the infrahyoid region has importance in surgical management of oral and oropharyngeal cancer [12].

Embryologically, the infrahyoid muscles develop from a muscle primordium in the anterior cervical area, which divides into a superficial layer and a deep layer. The deep layer develops to the sternothyroid and thyrohyoid muscles. The superficial layer becomes the splenius spreading in the cervical region, the intermediate part of which becomes degenerated in humans and the splenius is separated into the internal and external muscles. This internal muscle becomes the sternohyoid muscle and external muscle becomes the omohyoid muscle which runs obliquely in the lateral cervical area [23]. The existence of an additional muscle, cleidohyoid may be due to persistence of the fetal omohyoid and could be attributed to the primitive morphology of the splenius [9].

## Conclusion

Variation in origin, insertion, and morphology of the infrahyoid muscles including the cleidohyoideus accessorius have been reported in previous studies. However, herein, we report an unreported variant of cleidohyoid muscle. This variant muscle could be named as “sternocleidohyoid muscle” and to the best of our knowledge, there is no report on such a muscle in the literature. Knowledge of this muscle could be useful in surgical interventions and diagnostic procedures related to the anterior aspect of the neck.
